# Troponin elevation in acute ischemic stroke (TRELAS) - protocol of a prospective observational trial

**DOI:** 10.1186/1471-2377-11-98

**Published:** 2011-08-08

**Authors:** Jan F Scheitz, Hans-Christian Mochmann, Christian H Nolte, Karl G Haeusler, Heinrich J Audebert, Peter U Heuschmann, Ulrich Laufs, Bernhard Witzenbichler, Heinz-Peter Schultheiss, Matthias Endres

**Affiliations:** 1 Center for Stroke Research Berlin, Charité - Universitätsmedizin Berlin, 10117 Berlin, Germany; 2 Klinik für Neurologie, Charité- Universitätsmedizin Berlin, 10117 Berlin, Germany; 3 Medizinische Klinik für Kardiologie und Pulmonologie, Charité - Campus Benjamin Franklin, 12200 Berlin, Germany, Germany; 4 Klinik für Innere Medizin III, Kardiologie, Angiologie und Internistische Intensivmedizin, Universitätsklinikum des Saarlandes, 66421 Homburg, Germany

## Abstract

**Background:**

Levels of the cardiac muscle regulatory protein troponin T (cTnT) are frequently elevated in patients with acute ischemic stroke and elevated cTnT predicts poor outcome and mortality. The pathomechanism of troponin release may relate to co-morbid coronary artery disease and myocardial ischemia or, alternatively, to neurogenic cardiac damage due to autonomic activation after acute ischemic stroke. Therefore, there is uncertainty about how acute ischemic stroke patients with increased cTnT levels should be managed regarding diagnostic and therapeutic workup.

**Methods/Design:**

The primary objective of the prospective observational trial TRELAS (TRoponin ELevation in Acute ischemic Stroke) is to investigate the frequency and underlying pathomechanism of cTnT elevation in acute ischemic stroke patients in order to give guidance for clinical practice. All consecutive patients with acute ischemic stroke admitted within 72 hours after symptom onset to the Department of Neurology at the Campus Benjamin Franklin of the University Hospital Charité will be screened for cTnT elevations (i.e. >= 0.05 μg/l) on admission and again on the following day. Patients with increased cTnT will undergo coronary angiography within 72 hours. Diagnostic findings of coronary angiograms will be compared with age- and gender-matched patients presenting with Non-ST-Elevation myocardial infarction to the Department of Cardiology. The primary endpoint of the study will be the occurrence of culprit lesions in the coronary angiogram indicating underlying co-morbid obstructive coronary artery disease. Secondary endpoints will be the localization of stroke in the cerebral imaging and left ventriculographic findings of wall motion abnormalities suggestive of stroke-induced global cardiac dysfunction.

**Discussion:**

TRELAS will prospectively determine the frequency and possible etiology of troponin elevation in a large cohort of ischemic stroke patients. The findings are expected to contribute to clarify pathophysiologic concepts of co-morbid cardiac damage in ischemic stroke patients and also to provide a basis for clinical recommendations for cardiac workup of such patients.

**Trial registration:**

clinicaltrials.gov NCT01263964

## Background

Fighting heart disease and stroke is the agenda of the American Heart Association. What is more, the two diseases show intense connections as indicated by the high proportion of strokes with cardioembolic origin. Moreover, cardiac derangement has been observed in the context of acute neurological disorders causing myocardial dysfunction, arrhythmia, ECG alterations and rise of cardiac troponins [1,2].

The troponin protein complex consists of three subunits, namely troponin C, I and T, and contributes to the modulation of striated muscle contraction. Cardiac troponin C is identical to its skeletal isoform, but for troponin I and troponin T unique cardiac isoforms were identified. Monoclonal antibodies distinguish cardiac troponin T (cTnT) isoforms from fetal cardiac and skeletal isoforms without cross-reactivity, allowing the conclusion that elevation of cTnT in the blood stream always reflects myocardial injury [3,4]. However, the etiology of this myocardial injury is not revealed in the individual case, because increased cTnT was also found in the absence of thrombotic occlusion of a coronary vessel in other clinical conditions with cardiac injury or strain, such as heart failure, perimyocarditis, supraventricular tachycardias and pulmonary embolism [5,6]. Moreover, approximately 50% of the patients with renal insufficiency show increased cTnT values in the absence of symptoms of myocardial infarction [7]. This may relate to associated left ventricular (LV) hypertrophy, "silent" micro-infarctions or decreased renal troponin excretion [5,7].

Interestingly, increased cTnT was also reported to occur in 5-34% of patients with acute ischemic stroke [8]. In several studies, the elevation of cTnT was associated with stroke severity on hospital admission, insular cortex lesions, short- and long-term clinical outcome and increased risk of mortality [9-12], indicating prognostic significance of increased cTnT in acute ischemic stroke.

In the individual stroke patient the cause of troponin elevation is uncertain. One possible explanation in some patients may be the coincidence of acute coronary syndrome (ACS) leading to ischemic myocardial necrosis. This notion is supported by the fact that stroke and myocardial infarction share common risk factors and by the high prevalence of coronary artery disease (CAD) in stroke patients [13].

Neurogenic cardiac damage is another possible cause. Cardiac injury might result from autonomic imbalance after stroke affecting cortical areas controlling autonomic function, e.g. the insular cortex. A subsequent catecholamine surge may induce global LV dysfunction with ventriculographic findings of LV apical ballooning [14,15].

Due to the uncertainty regarding the underlying etiology of cTnT elevation in ischemic stroke patients the extent and time point of cardiologic work-up in this clinical situation is still under debate. Therefore, the aim of TRELAS is to determine the prevalence of troponin elevation in a prospective cohort of ischemic stroke patients and to display the frequency of underlying acute obstructive CAD, as defined by angiographic culprit lesions. Further objective is to clarify the frequency of associated neurocardiogenic LV dysfunction and insular cortex lesions.

## Methods/Design

### Study design and study population

TRELAS is a prospective observational, single centre, matched-pair controlled trial conducted by the Center for Stroke Research Berlin and the Department of Cardiology at the Campus Benjamin Franklin of the Charité University Hospital, Berlin. Patients with acute ischemic stroke and elevated cTnT, meeting the inclusion and exclusion criteria, as depicted in table 1, are eligible for the study. Approval by the local ethics committee (EA4/118/10) was obtained before trial registration in December 2010 (clinicaltrials.gov NCT01263964).

**Table 1:** TRELAS: Inclusion and exclusion criteria

Inclusion criteria
1. Acute ischemic stroke, confirmed by cerebral imaging
2. Inclusion within ≤ 72 hours after symptom onset
3. hsTroponin T ≥0.05 μg/l

**Exclusion criteria**

1. Impaired renal function (creatinine >1,2 mg/dl)
2. Limited life expectancy or premorbid mRS ≥ 4
3. Contraindications for coronary angiography
4. Age < 18 years
5. Pregnancy
6. Patient unwilling to give informed consent
7. ST- elevation myocardial infarction

### Procedures

All consecutive patients with imaging-confirmed ischemic stroke admitted within 72 hours after symptom onset to the Department of Neurology will be screened for increased cTnT immediately on admission and the following day. In the case of cTnT elevation (i.e. >= 0.05 μg/l) and normal serum creatinine (<= 1.2 mg/dl) coronary angiography (CAG) will be carried out within the next 72 hours. In the absence of culprit lesions, which are defined as complex coronary stenoses as suggested by Ambrose [16,17], further treatment will not include dual antiaggregation. If a culprit lesion is present in the coronary angiogram, coincident myocardial infarction is regarded to be the cause of cTnT increase. Further intervention will depend on individual risk-benefit analysis. Possible complications of CAG during the study were discussed with the local ethics committee and will be documented accurately. Subsequent to CAG patients' vital signs, physical status, urinary excretion and peripheral pulse status will be monitored by experienced physicians. Monitoring will be done in the 12-bed Stroke Unit or on the cardiologic Intensive Care Unit, respectively.

Age- and gender-matched patients with Non-ST-elevation-ACS (NSTE-ACS), thus showing elevated troponin levels above 0.05 μg/l *per definitionem*, will serve as controls to allow comparison with regard to frequency of culprit lesions and left ventricular (LV) dysfunction. All patients admitted to the Department of Cardiology, Campus Benjamin Franklin with NSTE-ACS who undergo early CAG within 72 hours and who give informed consent for participation will be eligible as control group.

**Figure 1:**
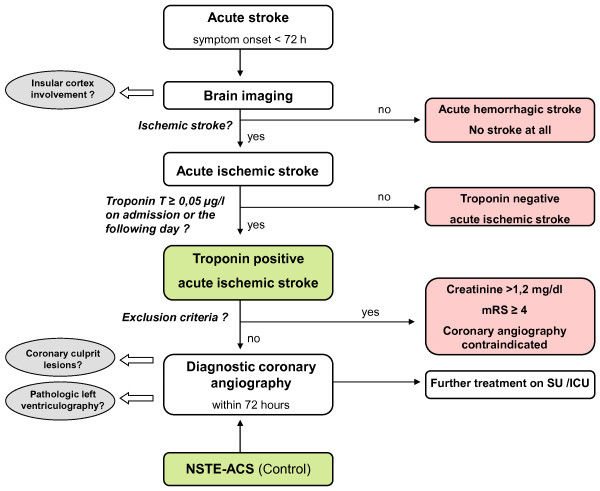
**Flow chart of study procedures**. Gray ellipses: Outcome measures, Green boxes: Study population, Red boxes: Excluded population; Abbreviations: ICU: Intensive Care Unit, mRS: modified Rankin Scale, NSTE-ACS: Non-ST-elevation acute coronary syndrome, SU: Stroke Unit.

A flow chart of study procedures is depicted in Figure 1. Diagnostic procedures and interpretation are described in more detail in the respective section further below.

#### Medical history and neurological examination

After recording of patient characteristics (age, gender, stroke risk factors including arterial hypertension, hypercholesterolemia, heart failure and atrial fibrillation, history of coronary artery disease, chest pain according to the Braunwald Classification [18], nicotine and alcohol consumption and known malignant tumors) neurological examination will be carried out by certified investigators using the National Institute of Health Stroke Scale (NIHSS) and modified Rankin scale (mRS). The NIHSS is a validated tool used in many clinical trials to evaluate stroke severity and graduate neurological deficits as well as alterations during the patients' hospital stay [19,20]. The mRS is a validated instrument to measure the degree of disability [21,22]. Patients will be scored on admission and on day 7 or on hospital discharge, which is typically between day 4 and day 10 after hospital admission, respectively. Data on demographic information, course of symptoms and stroke etiology, classified by the TOAST classification [23], will be recorded according to the German Stroke database registry guidelines.

#### Laboratory measures

Serum cTnT levels are determined on admission using a high sensitive Troponin T assay (Roche Elecsys^® ^Troponin Ths, Mannheim, Germany) with an upper reference limit for the 99^th ^percentile of the normal reference population at 0.014 μg/l and a coefficient of variation of less than 10% at 0.013 μg/l [24]. Our laboratory defined a cut-off value of 0.05 μg/l to establish the diagnosis of NSTE-ACS. This value is regarded to be equivalent to 0.03 μg/l, the cut-off value of fourth generation cTnT assays [25]. If cTnT is below the cut-off on admission a second blood sample will be obtained on the following day. Further blood markers, including creatinine, are determined as a part of routine laboratory measures on admission and in the course of the hospital stay.

#### Coronary angiography and further cardiologic work-up

CAG will be performed by trained cardiologists according to local guidelines. The respective CAG procedure itself as well as the usage of specific devices will be left to experience and expert knowledge of the cardiologist. To determine coronary status both coronary arteries will be visualized. Complex coronary stenoses showing irregular borders, ulceration or filling defects suggestive of the presence of intracoronary thrombus will be defined as culprit lesions as suggested by Ambrose [17]. Furthermore, left ventriculography will be performed to record wall motion abnormalities, especially LV apical ballooning [26]. Patients will be informed about procedure-related risks a second time independently by the cardiologist performing the examination. All patients will undergo routine diagnostic work-up including standard 12-lead ECG, Holter-ECG and transesophageal ultrasound according to recommendations of the German Stroke Society [27].

#### Cerebral imaging

Brain imaging will be performed according to ongoing institutional protocols. Whenever possible imaging will be done by using 3T MRI (Tim Trio, Siemens AG, Erlangen, Germany) [28]. Otherwise, cerebral computed tomography (64-slice, Siemens AG, Erlangen, Germany) will be used. Stroke localization will be determined by experienced neuroradiologists blinded to clinical details. Findings will be dichotomized into absence or presence of insular cortex involvement. Furthermore, the affected brain circulation territory will be specified as right anterior, left anterior or posterior circulation.

### Hypotheses and outcomes

The primary hypothesis of TRELAS is that cTnT elevation in acute stroke patients is generally not caused by coincidental myocardial infarction. Thus, we expect angiographic culprit lesions to occur less frequently in patients with acute stroke and elevated cTnT than in patients with NSTE-ACS.

Furthermore, we hypothesize that strokes affecting the insular cortex can be found more often in patients with increased cTnT than with normal cTnT. A further secondary hypothesis is that LV global cardiac dysfunction occurs more frequently in stroke patients with increased cTnT than in patients with NSTE-ACS. Therefore, the findings of the left ventriculography will serve as an outcome measure.

### Power calculation and sample size estimates

A power calculation was conducted based on literature review describing the frequency of culprit lesions in 85% of the patients with NSTE-ACS [29] and an estimated occurrence of culprit lesions in 35% of stroke patients with troponin elevation. We aim to include 58 patients, 29 patients with acute ischemic stroke and 29 age- and gender-matched patients with NSTE-ACS, to detect the estimated difference of 50% regarding the occurrence of angiographic culprit lesions within the matched-pairs with a statistical power of 90% at a significant level of 5%. This analysis included an estimated patient drop-out rate of 20%.

## Discussion

Elevated troponin levels are assumed to occur in up to 1 out of 6 patients presenting with acute cerebral ischemia and are associated with stroke severity, unfavorable short- and long-term clinical outcome [2,8]. Two candidate mechanisms are regarded to be relevant for troponin elevation in acute ischemic stroke when renal insufficiency is excluded. First, coincident ACS may lead to focal ischemic myocardial necrosis by means of thrombotic occlusion of a coronary vessel. Second, stroke-induced autonomic imbalance with subsequent surge of catecholamines could induce global damage and dysfunction of myocardial tissue and the release of cTnT. However, the underlying cause of cTnT elevation in the individual stroke patient remains questionable, leading to a diagnostic and therapeutic dilemma for the attending physician. In order to rule out coincident ACS reliably coronary status has to be clarified. However, estimated risks of cerebral bleeding complications limit the diagnostic work-up using CAG in patients with acute stroke.

Previous studies investigating troponin elevations in patients with ischemic stroke mainly described the prevalence and prognostic significance but data concerning co-existing acute CAD are scarce. Myocardial perfusion scintigraphy was carried out by Jensen et al. in 11 acute stroke patients with elevated cTnT, a cohort too small to produce significant results [11]. So far, no study has used CAG to determine coronary status in patients with elevated cTnT and acute cerebral ischemia.

All stroke patients included in TRELAS will undergo CAG in order to determine the frequency of coronary culprit lesions associated to cTnT elevation. CAG is regarded to be the diagnostic gold standard for establishing the diagnosis of myocardial infarction [30,31]. Although non-invasive coronary CT imaging substantially improved over the last years, current guidelines recommend CAG in a clinical situation suggestive of myocardial infarction [30]. Moreover, using CAG enables therapeutic intervention if a culprit lesion was identified. Within our study CAG will be done within 72 hours as recommended by guidelines of the American Heart Association and European Society of Cardiology for patients with NSTE-ACS and high likelihood of CAD [30,31] because patients with NSTE-ACS seem to benefit from early CAG [32]. We have not defined typical chest pain as an inclusion criteria because one third of all myocardial infarctions in the elderly remain clinically "silent" [33,34]. In fact, cardiovascular disease is the most common cause of death in one-year stroke survivors [35] and a substantial amount of stroke patients have asymptomatic coronary stenoses [13] indicating CAD.

A reason for not performing CAG in stroke patients are safety concerns with an increased risk of brain hemorrhagic transformation and parenchymal hemorrhage because of anticoagulation during the procedure. However, only small amounts of heparin, if any, need to be administered during CAG and the increased risk of bleeding is rather caused by double-antiaggregation following application of coronary stents. In a recent study, CAG was performed in order to show previously unknown CAD in a large cohort of patients with ischemic stroke and proved to be safe even in the early phase of acute stroke (6-11 days after onset). In 315 stroke patients undergoing CAG no procedural adverse event was observed except one groin hematoma [13].

A further objective of the TRELAS study is to investigate whether there is a correlation between troponin elevation and ischemia in the insular cortex which is regarded to play a crucial role in controlling the autonomic nervous system [36]. In particular, right-sided insular lesions have been linked to disturbance of autonomic balance [37]. Interestingly, Ay et al. conducted a voxel-wise analysis of diffusion weighted imaging lesions and reported a significant association of right insular cortex involvement and cTnT elevation [12]. However, Barber et al. found no association of insular cortex stroke and increased serum cTnT [38]. We hope the results of TRELAS to contribute to this issue.

Another aim of TRELAS is to assess the relation between neurogenic cardiac dysfunction and troponin elevation in ischemic stroke patients. Several studies reported disturbance of autonomic cardiac function with LV wall motion abnormalities in patients with acute ischemic stroke or subarachnoidal hemorrhage, respectively [14,39,40]. In this context a unique kind of cardiomyopathy, termed *Takotsubo cardiomyopathy *(TTC), seems noteworthy. Excessive catecholamine levels in the context of acute stressful events cause LV dysfunction with LV apical ballooning in TTC [41], frequently combined with cTnT elevation despite of missing CAD [26]. Diagnosis of TTC can be established via CAG and left ventriculography because the cardiac wall motion abnormalities associated with TTC can usually be shown and concomitant obstructive CAD can be ruled out simultaneously [26]. The results of TRELAS will contribute to the question whether cTnT elevation in ischemic stroke is associated with myocardial LV dysfunction, and whether TTC is a relevant differential diagnosis in this clinical situation.

Impaired renal function is an exclusion criteria because diagnostic sensitivity to detect ACS is decreased in patients with renal failure [42] and those patients are prone to adverse effects of the contrast agents administered for CAG. Nevertheless, only a few studies with troponin elevation in stroke excluded patients with renal insufficiency [12,15,43].

To properly detect elevations of cTnT a high-sensitive troponin assay will be used. The assay was established recently to improve diagnostic precision, which is necessary according to the 2007 consensus definition of acute myocardial infarction [44]. We do not expect significant inclusion of "false positive" patients in TRELAS because of the chosen cut-off, which is equal to cut-offs of former fourth cTnT assays [25] and the exclusion of patients with impaired renal function.

There are some limitations of our study. First of all, CAG is conducted in a rather small cohort of 29 stroke patients. This sample size is adjusted to show a 50% difference in the occurrence of coronary culprit lesions between the matched-pairs. Thus, smaller differences are not detectable with a respective statistical power. Second, power estimation was carried out for finding significant results concerning the primary endpoint. Therefore, it might not be possible to present significant findings concerning the secondary endpoints. Third, the cerebral imaging modality will probably be heterogeneous in our study sample due to impossibility to ensure MRI in each stroke patient.

Taken together, troponin elevation is a frequent observation in patients with acute ischemic stroke and is associated with adverse outcome. As the underlying mechanism in the individual case is unclear there is a considerable diagnostic and therapeutic uncertainty. A focal cardiac ischemia due to acute rupture of coronary plaques requires urgent initiation of invasive cardiac diagnostics and intensified therapy. However, a proven stroke-mediated cardiomyopathy would open doors for heart protection strategies. The observational TRELAS trial will describe the prevalence of troponin elevation in a prospective cohort of acute ischemic stroke patients and will determine the frequency of possible etiological causes. Our findings may provide a basis for further recommendations regarding the extent and time point of cardiac work-up in this clinical setting.

## List of abbreviations

ACS: acute coronary syndrome; CAD: coronary artery disease; CAG: conventional invasive coronary angiography; cTnT: cardiac Troponin T; ECG: electrocardiography; LV: left ventricular; MRI: Magnet Resonance Imaging; mRS: modified Rankin scale; NIHSS: National Institute of Health Stroke Scale; NSTE-ACS: Non-ST-elevation acute coronary syndrome; TOAST: Trial of Org 10172 in Acute Stroke Treatment; TRELAS: TRoponin ELevation in Acute ischemic Stroke; TTC: Takotsubo Cardiomyopathy.

## Competing interests

The authors declare that they have no competing interests.

## Authors' contributions

All authors approved the final manuscript and contributed to its revision. CHN, BW and ME conceived the idea and together with JFS, HCM and BW conducted the design of the trial. CHN, HCM, ME, JFS, KGH, UL, HJA and HPS were involved in further planning. JFS drafted the manuscript. JFS, KGH, CHN and HCM will recruit patients. ME, HJA and HPS will participate in the supervision of the trial. PUH did and will do the statistical calculations.

## Pre-publication history

The pre-publication history for this paper can be accessed here:

http://www.biomedcentral.com/1471-2377/11/98/prepub
